# Comments on “Efficacy and safety assessment of acupuncture and nimodipine to treat mild cognitive impairment after cerebral infarction: a randomized controlled trial”

**DOI:** 10.1186/s12906-017-1629-z

**Published:** 2017-02-20

**Authors:** Jose M. Moran, Juan D. Pedrera-Zamorano

**Affiliations:** University of Extremadura, Nursing Department, Metabolic Bone Diseases Research Group, Health Sciences Research Methodology Collaboration Group, Avd. Universidad s/n, 10001 Cáceres, Spain

We read with great interest the article reporting on the efficacy and safety of acupuncture and nimodipine therapy for the treatment of mild cognitive impairment after cerebral infarction [1]. We would like to highlight that the article by Wang and colleagues reports outcomes that differ from those initially described [2]. The article reference the pre-specified primary outcomes (efficacy) and only one (Montreal Cognitive Assessment, “MoCA”) of the six pre-specified secondary outcomes. The CONSORT guidelines on the best practice in a trial reporting [3] are implemented to reduce the risk of selective outcome reporting and require that all pre-specified primary and secondary outcomes be reported.

Additionally, we would like to highlight the inappropriate use of statistical tests performed in this manuscript. The analysis performed does not make justice to the considerable effort realized by the authors in this manuscript. Although reanalyzed, based in the supplementary data provided, there were no major changes to the results presented by them; but, such an inappropriate use of statistics should never pass the peer review process and BMC Complementary and Alternative Medicine should notice that.

The authors indicate that “*For intra-group comparison of MoCA score improvement at the end of 3-month therapy* versus *that at the follow-up, Kruskal-Wallis rank sum test was used for the acupuncture alone group; independent sample t-test was used for the nimodipine alone and nimodipine + acupuncture groups. Kruskal-Wallis rank sum test was used for comparison of percentage improvement of MoCA score at the end of 3-month therapy* versus *that at the follow-up in each group*”. The appropriate statistical test for analyzing two groups in a longitudinal study is not the Kruskal–Wallis test; if you want a non-parametric test, please use the Wilcoxon signed-rank test because under the same treatment, MoCA measurements are dependent between baseline, at the end of treatment and at the follow-up. The Kruskal-Wallis rank sum test is intended for unrelated samples (for example, inter-group comparisons and always more than two groups). The use of an “*independent sample t-test*” is also inappropriate for intra-group comparisons because measurements are *dependent*, as patients are always the same at baseline, at the end of treatment and at the follow-up. Additionally, the nimodipine alone data is not normally distributed (*P* < 0.001 in the Kolmogorov-Smirnov test at baseline, at the end of 3-month treatment, and at the follow-up); therefore, a parametric test such as the *t*-test should not be used. Similarly, in the nimodipine + acupuncture group, neither baseline (*P* = 0.026 in the Kolmogorov-Smirnov test) nor the follow-up (*P* = 0.030 in the Kolmogorov-Smirnov test) data are normally distributed; therefore, a parametric test cannot be used. Alternatively, authors should use the Wilcoxon signed-rank test for the two intra-group comparisons.

Inter-group comparisons, when performed between two groups, are also incorrectly used; an appropriate test would be the Mann–Whitney *U* test or a similar test, not the Kruskal-Wallis rank sum test, which is intended for use when there are more than two groups.

Using the appropriate statistical test, the mean MoCA score increased significantly (*P* < 0.0001) among all the comparisons at the end of the 3-month treatment compared to baseline values, and a further increase that remained significantly higher at the 3-month follow-up was observed, as the authors indicated. Additionally, the mean percentage of MoCA improvement at the follow-up was higher than that at the end of the 3-month treatment under all treatments (*P* = 0.022 for nimodipine alone, *P* < 0.001 for acupuncture, and *P* < 0.001 for nimodipine + acupuncture). These results suggest that all 3 therapies may improve cognitive function under the studied circumstances.

However, MoCA scores at the follow-up did not differ significantly between the acupuncture alone group (26.1 ± 3.6) compared to the nimodipine alone group (24.2 ± 4.6) (*P* = 0.055), and there were no statistically significant differences between the nimodipine + acupuncture MoCA score (26.0 ± 2.8) and that of the nimodipine alone group (*P* = 0.086). Similar results were observed at the end of the treatment period; therefore, no significant differences were found in the inter-group comparisons with respect to the MoCA score.

Although reanalyzed data using the appropriate statistical tests did confirm some of the data presented by the authors, it is questionable that such results indicate that the combinatory therapy of nimodipine and acupuncture shows superior efficacy on post-cerebral infarction MCI to nimodipine monotherapy and acupuncture monotherapy. Clinical relevance is not automatically evidentiated if there is statistical significance. The cause(s) may be that the sample is too small and/or the dispersion is too high. Thus, a decision for clinical significance under the study circumstances on the basis of the *P*-value alone may be simplistic.﻿
